# Study protocol for PRISE: a longitudinal study of sexual harassment during the transition from childhood to adolescence

**DOI:** 10.1186/s40359-019-0345-5

**Published:** 2019-11-12

**Authors:** Therése Skoog, Kristina Holmqvist Gattario, Carolina Lunde

**Affiliations:** Department of Psychology, University of Gothenburg, Gothenburg, Sweden

**Keywords:** Sexual harassment, Peer victimization, School, Longitudinal, Late childhood, Adolescence, Developmental transition

## Abstract

**Background:**

Sexual harassment is a widespread problem with serious consequences for individuals and societies. It is likely that sexual harassment among peers has its main onset during the transition from late childhood to early adolescence, when young people enter puberty. However, there is a lack of systematic research on sexual harassment during this developmental period. Thus, there is very little information about the prevalence of sexual harassment during this important transition, its consequences, and how to effectively intervene against and prevent the problem. The primary objective of the described project, entitled Peer Relations In School from an Ecological perspective (PRISE), is to examine sexual harassment and its developmental correlates during the transition from late childhood to early adolescence.

**Methods:**

The PRISE study has a longitudinal design over 3 years, in which a cohort of children (*N* = 1000) and their main teachers (*N* = 40) fill out questionnaires in grades 4, 5, and 6. The questionnaires assess aspects of peer sexual harassment and potential correlates including biological (e.g., pubertal development), psychosocial (e.g., self-assertiveness, self-image, peer relations), and contextual (e.g., classroom climate, norms) factors. In addition, we will examine school readiness and policies in relation to sexual harassment and collect register data to assess the number of reports of sexual harassment from the participating schools.

**Discussion:**

The PRISE study will enable the researchers to answer fundamental, unresolved questions about the development of sexual harassment and thus advance the very limited understanding of sexual harassment during the transition from childhood to adolescence - a central period for physical, sexual, and social development. Due to the sensitive nature of the main research concepts, and the age of the participants, the ethical aspects of the research need particular attention. Ultimately, the hope is that the PRISE study will help researchers, policy makers, and practitioners develop, and implement, knowledge that may help in combating a major, current societal challenge and adverse aspect of young people’s developmental ecologies.

## Background

Sexual harassment can be defined broadly as “improper behavior that has a sexual dimension” [1] or “unwanted sexual attention” [2]. It includes a range of verbal, physical, and visual direct or indirect behaviors that the recipient perceives as unwelcome and/or unwanted. Some examples are uninvited sexual comments, grabbing, touching, and requests for sexual favors. As opposed to legal definitions of sexual harassment, psychological definitions emphasize the victim’s subjective experience when determining whether an act should be regarded as sexual harassment or not [3].

Testimonies from the #metoo movement, and evidence from a small, tentative body of mainly cross-sectional research, converge to reveal that sexual harassment becomes part of young people’s lives early in their development. It is well-established that the problem is highly prevalent in early adolescence [4–7]; however, research on sexual harassment in late childhood (ages 10–12 years), is still in its infancy. This is unfortunate considering that late childhood is a period that is central for general physical, sexual, and social development, and, importantly, the transition from childhood to early adolescence has been pointed out as the time in life when children are typically confronted with and start engaging in peer sexual harassment for the first time. Trigg and Wittenstrom [8], for example, reported that 15% of high school and college aged students recalled being sexually harassed in the first through fifth grades. Moreover, in one study, more than 90% of students in middle school in the US (mean age 12.5 years) reported having been the target of some form of sexual harassment the previous school year, with verbal harassment (e.g., name calling) being the most common form of sexual harassment [9]. Despite the evidence suggesting a high prevalence of sexual harassment at early ages, and the fact that knowledge of the developmental processes underlying sexual harassment among young people is needed for effective prevention, the current literature lacks comprehensive, developmentally and ecologically informed longitudinal studies covering the transition from late childhood to early adolescence. This is surprising not least given that the pubertal process, which occurs during this period for most girls and boys [10], has been identified as a main trigger for the onset of sexual harassment [2, 11, 12]. More studies that follow young people over the course of the transition from late childhood to early adolescence are needed, preferably starting before puberty and its associated marked rise in sexual harassment.

### What is known about the development of sexual harassment at young ages?

The existing literature on sexual harassment among young people (primarily adolescents) has provided some important insights into the phenomenon and its consequences. Firstly, studies from different countries in Europe, North America, Asia, and Australia consistently find high prevalences of sexual harassment victimization among adolescents. In many studies [7, 13–15], half or more than half of the adolescents report being the targets of sexual harassment. These findings clearly indicate that sexual harassment is a significant and universal problem among young people around the globe.

Secondly, adolescent research has identified a number of individual characteristics that are linked to an increased risk of sexual harassment victimization and perpetration. Concerning gender, sexual harassment has been described traditionally as harm that men or boys expose women or girls to [16, 17]. Accordingly, most adolescent research has demonstrated that girls are more often the targets of, and boys more often the perpetrators of, sexual harassment [2, 5, 18–20]. In sharp contrast, however, other findings have revealed that boys are more exposed to direct sexual harassment than girls [7, 21, 22]. Research that examines specific forms of sexual harassment in relation to gender [19] has found that for some forms, girls are more exposed (e.g., being touched, grabbed or pinched in a sexual way) and for others, boys are more exposed (e.g., homophobic name calling). Another set of studies have found that sexual harassment occurs both within and between both sexes and in both directions [13, 23, 24]. Other individual characteristics that have been related to sexual harassment in adolescence include pubertal timing [25, 26], gender-role contentedness [27], and sexual behavior [12, 25, 26].

Thirdly, in addition to individual characteristics, a body of literature has identified environmental characteristics that are related to sexual harassment. In addition to e-contexts [28], educational settings have been identified as a major arena for sexual harassment among young people [19, 29]. Some of the existing studies have found that certain aspects of the school context, including teacher maltreatment [22] and feeling disconnected from school [24], are linked to a higher prevalence of sexual harassment. Aspects of the peer context, including bullying and peer relationship problems [22], having peers with problematic behavior [30], participation in mixed-gender peer groups [2], and romantic relationship status [24] have also been linked to sexual harassment. This is also true for aspects of the parent-adolescent relationship [31].

Finally, studies have identified an array of negative consequences of sexual harassment. Interestingly, and perhaps surprisingly, research has found that early adolescents seem to view verbal harassment as the most upsetting forms of sexual harassment victimization [19]. Some of the harms of sexual harassment among young people include lower self-esteem, poor physical and mental health, and trauma symptoms [11], shame, poor body image [14], depressive symptoms [18, 32], substance use [33], adjustment problems [30], and academic problems [34]. Only a few protective factors against these consequences have been identified, including higher self-esteem and higher perceived support from others [35]. Considering that the negative effects have been found in different domains of functioning, sexual harassment appears to have a pervasive, negative influence on young people’s development. Taken together, the existing findings point towards the importance of early interventions, as early as before or around the advent of puberty, to combat the problem of sexual harassment among young people. For these interventions to be effective, understanding how sexual harassment develops at young ages is a fundamental first step. This step is yet to be taken.

### What is unknown about the development of sexual harassment in young ages?

Although important knowledge has been gained from the growing literature on sexual harassment among young people, there are fundamental, unresolved questions that remain to be answered. More research that can answer these questions is urgently needed, given the high prevalence and the adverse consequences associated with sexual harassment. One central limitation in the literature is that most existing studies focus on adolescence; few focus on late childhood. Therefore, little is known about sexual harassment and its development in late childhood and the transition to early adolescence. Furthermore, the vast majority of studies are cross-sectional; few are longitudinal. The lack of prospective, longitudinal studies hinders insight into the developmental processes that underlay peer sexual harassment, its correlates, and consequences over time. Given that puberty is an assumed trigger of sexual harassment [2, 11, 12], research that aims to fully understand the developmental processes related to sexual harassment and its developmental consequences should commence at or even before puberty (i.e., in late childhood). Such studies are important not only to find out the prevalence of sexual harassment at different ages, but also because developmental processes could be different at different ages. For instance, it is possible that the consequences of sexual harassment at early ages (i.e., late childhood) could be different, and perhaps even worse, compared to later ages (e.g., late adolescence), given that younger children may be less skilled in coping with situations of sexual harassment. To date, whereas there is a body of research on sexual harassment over the course of early to late adolescence [36], studies that prospectively follow a substantial group of children from late childhood through the transition to early adolescence (i.e., ages 10–13) are missing from the literature.

Another limitation in the literature is that few studies have taken an ecological approach to the study of sexual harassment among young people. The focus in previous studies has primarily been on the individual level. Similarly, studies have primarily relied on data from single informants (i.e., typically victims of sexual harassment). Research needs to pay attention to the ecological context, on multiple levels, in which sexual harassment takes place, develops, and affects young people. One of the most central developmental arenas for young people is school, but at the same time, school has also been identified as a major arena for sexual harassment among young people [29]. Worryingly, studies further indicate that many schools fail to adequately acknowledge and combat sexual harassment in school [37, 38]. This is troublesome given that sexual harassment may interfere with children’s possibilities “to receive an equal educational opportunity” [39], which is also mirrored in findings identifying negative consequences such as absenteeism and lowered grades following sexual harassment [34]. In line with a developmental-ecological perspective [40], and in order to address the problem of sexual harassment efficiently, school-based studies that gather information from different informants are warranted. This would help to further knowledge about how, for example, attitudes and norms at the school and classroom levels affect the prevalence of sexual harassment. It would also enable an increased understanding for the barriers that may discourage young people’s disclosure of sexual harassment.

### Theoretical framework of the PRISE study

Against this background, this study protocol describes a new longitudinal, ecologically informed research program in Sweden, aiming to address sexual harassment among peers through the transition from late childhood to early adolescence: the Peer Relations In School from an Ecological perspective (PRISE) study. The current project has been designed to address and overcome the shortcomings in the current literature concerned with sexual harassment in early development.

Several models of sexual harassment have previously been explored in the literature. The PRISE study is framed within developmental-contextual theoretical perspectives on sexual harassment [2]. A key assumption in the current project is that individuals’ experiences of sexual harassment are embedded in their environmental context. Thus, a key theoretical framework for this project is the developmental-ecological perspective [40]. In line with this, the occurrence of sexual harassment in school can be seen as a result of the interaction between the individual and his or her (school) context. This notion is in line with a small body of literature that suggests that factors which protect against homophobic bullying include a positive school climate [41]. In the described project, we examine the interaction between three layers or levels of the individual and his or her context: the individual level, the classroom level, and the school level. At each level, there may be risk factors, protective factors, and potential consequences related to the occurrence of sexual harassment. The individual level includes individuals’ own experiences of being harassed, harassing others, or witnessing harassment. It also includes biological (e.g., gender, pubertal development) and psychological (e.g., self-esteem, body esteem, resilience, satisfaction with class and school, reactions if sexually harassed) factors within the individual. The classroom level includes the occurrence of sexual harassment in the class. It also includes teachers’ thoughts about sexual harassment (e.g., the seriousness of it), their efficacy in handling situations of sexual harassment in the classroom, and how peers react to sexual harassment in the class. The school level involves the occurrence of sexual harassment in the school, interventions conducted in the school, and school readiness to handle sexual harassment. Considering sexual harassment as a result of the interaction between these levels, individuals’ development can be influenced not only by their own experiences of being harassed or harassing others, but also by situations of sexual harassment in their peer group (and how they are handled by their teacher) and at their school. Research has shown that teachers have more knowledge about bullying than they do about sexual harassment [42], which may lead to teachers not seeing situations of sexual harassment, nor understanding their vital role in counteracting them.

The PRISE study is further guided by the developmental theory of embodiment (DTE) [43]. This theory is helpful in outlining the possible processes involved in the relationship between being sexually harassed and the negative outcomes examined in this project (e.g., depressive symptoms and disordered eating). The DTE derives from social constructivist and feminist frameworks and explains how individuals’ – primarily girls’ and women’s – experiences of their bodies, i.e., *embodiment,* are shaped as they engage with the world. According to the theory, social experiences shape individuals’ embodiment via three core pathways: 1) the *physical* domain, 2) the *mental* domain of social discourses and expectations, and 3) the *social power and relational connections* domain. We suggest that sexual harassment may compromise individuals’ experiences of their bodies within all three domains. The physical domain concerns individuals’ experiences of ownership and agency in relation to their bodies. Experiences undermining body ownership and agency, such as having one’s body unwillingly scrutinized, commented on, or touched, by others, can inhibit experiences of embodiment. The mental domain of social discourses and expectations involves individuals’ experiences of stereotypes and expectations. Being exposed to disseminated stereotypes, for example stereotypical, restraining expectations regarding how girls and boys should behave, also undermines embodiment. The third domain of social power and relational connections includes experiences of empowering or disempowering relationships. Prejudicial treatment, harassment, and living in communities with gender inequality are among the experiences undermining embodiment within this domain.

We suggest that sexual harassment may undermine individuals’ experiences of their bodies within all three domains (the physical, mental, and social power and relational connections domain), and that this may lead to lower body esteem and lower psychological well-being. To our knowledge, the only longitudinal study examining sexual harassment among girls in late childhood (mean age 11.5 years) found that exposure to sexual harassment predicted a higher risk of developing disordered eating four years later [44]. Other studies have further established that low body esteem is a gateway to disordered eating as well as depression in adolescent girls [45]. Therefore, in line with the DTE, low body esteem is suggested to mediate the relationship between sexual harassment and its negative outcomes such as depressive symptoms and disordered eating.

In relation to the issue of gender, it should be noted that since past studies have shown that both girls and boys are sexually harassed by both girls and boys [23], we do not define sexual harassment as male sexual aggression against women. However, some studies lend support to the notion that sexual harassment may be more damaging for girls than for boys [46]. Nonetheless, this conclusion may be premature, as outcomes typically have been biased towards internalizing symptoms, which are more commonly reported by girls. In fact, some show that the link between sexual harassment and adjustment is stronger for boys than for girls [47]. Thus, it is possible that boys who are exposed to sexual harassment suffer equally damaging effects – but these effects may not have been captured by studies to date. The current project will examine a broader range of possible negative outcomes of sexual harassment, including externalizing symptoms which are typically more prevalent among boys.

### Research objectives

In summary, fundamental and urgent questions remain concerning the development of peer sexual harassment during the transition from childhood to adolescence [37]. The current literature lacks comprehensive, developmentally and ecologically informed longitudinal studies covering the transition from late childhood to early adolescence. The lack of studies of the years in which sexual harassment emerges is concerning given that it prevents a full understanding of its prevalence, consequences, and risk and protective factors. This understanding also needs to consider the different roles involved in sexual harassment (e.g., victims, perpetrators, and peers who witness it), and to be informed by ecological perspectives. Against the backdrop of the identified gaps in knowledge in the literature, the primary objective of the PRISE study is to examine the prevalence of sexual harassment over the course of the transition from late childhood to early adolescence and its developmental correlates among boys and girls. The study is developmentally informed and has a developmental-contextual approach, meaning that biological, psychological, social, and contextual factors will be examined in relation to sexual harassment over the course of three years (grades 4–6, ages 10–12 years).

The project is guided by four research questions:

1. What is the prevalence of peer sexual harassment (victimization, perpetration, and witnessing) during the transition from childhood to early adolescence (ages 10–12 years)?

2. What are the predictors of peer sexual harassment victimization, perpetration, and witnessing during the transition from childhood to early adolescence (ages 10–12 years)?

3. What are the developmental consequences of peer sexual harassment for different subgroups of children during the transition from childhood to early adolescence (ages 10–12 years)?

4. What biological, psychological, social, and contextual risk and protective factors moderate the potential link between sexual harassment and its developmental consequences during the transition from childhood to early adolescence (ages 10–12 years)?

## Methods/design

### Research design

This research project, designed to examine sexual harassment among peers during the transition from late childhood to early adolescence in Sweden, has a three-year longitudinal design. Data will be collected annually in grades 4 (T1) to 6 (T3) from one cohort of students, teachers, and schools via questionnaires. The questionnaires will cover aspects of sexual harassment experiences among peers as well as biological, psychological, social, and cultural factors that there are theoretical and/or empirical reasons to think might be related to sexual harassment. The study will run from 2019 to 2021.

### Participants

The research project will be conducted in Sweden. It may appear paradoxical that Sweden has a high level of gender equality [48] and at the same time a high prevalence of sexual harassment [49]. Therefore, Sweden provides an interesting and important context for the study of the development of sexual harassment in early development and its correlates [38].

The study will include 1000 male and female students in approximately 40 classes. About 25–30 Swedish public and private middle schools (mellanstadieskolor) will be recruited for the data collection. Most prior studies of sexual harassment have relied on data from single informants (i.e., typically victims of sexual harassment). The current project takes a developmental-ecological approach by collecting data from different informants at different ecological levels. For each of the participating classes, one main teacher will be included in the study and answer questions about the class and the school culture (*N* = 40). In addition, existing documentation will be collected from each school (*N* = 25–30). The sample size, *N* = 1000, is based on conventional calculations [50], aiming for 80% power, .05 alpha, the ability to detect small effect sizes, and using more than ten predictors (Miles & Shevlin, 2001). The size further accounts for some attrition (10%) that might occur over the study period.

### Measures

Table 1 provides an overview and brief description of the instruments that will be used in the study. The instruments have been chosen based on the theoretical framework [2, 40, 43] and prior empirical studies [4, 5, 13, 18]. Accordingly, data will be collected at the individual level (e.g., from student self-reports), classroom level (e.g., from teacher reports), and school level (e.g., from school data).

**Table 1:** Overview of the study instruments

Measure/Instrument	Description/Construct	Timing (T) of administration
*Student self-reports*		
Demographic information	Questions are asked about age, gender, living situation (housing and family structure), country of birth (own and parents’), language spoken at home, and socioeconomic status	T1, T2, T3^a^
Pubertal Developmental Scale [51]	5 items measuring pubertal status, pubertal timing. 1 item measuring perceived pubertal timing. 2 items measuring height and weight.	T3 Perceived pubertal timing at T1, T2, T3
The Child and Adolescent Social Support Scale [52]	12 items measuring emotional support from teachers, parents, classmates, and friends .	T1, T2, T3
Body Esteem Scale for Adolescents and Adults [53]	10 items measuring appearance-based body esteem	T1, T2, T3
Strengths and Difficulties Questionnaire [54]	10 items from the subscales measuring emotional and conduct problems	T1, T2, T3
Victim Scale [55]	5 items measuring general peer victimization (physical, verbal, social)	T1, T2, T3
School satisfaction (adapted, [56])	2 items, e.g., “Do you enjoy school?”	T1, T2, T3
Classroom satisfaction (adapted, [57])	4 items, for example, "We help each other"	T1, T2, T3
Sexual harassment victimization	6 + 5 items: verbal, visual, and physical sexual harassment victimization	6 items at T1 and T2 and 11 items at T3
Location	10 items: Location at school where the harassment took place	T1, T2, T3
Offender	5 + 4 items: Gender and age of perpetrator(−s)	T1, T2, T3
Witnesses	7 items: Witnesses to the harassment	T1, T2, T3
Disclosure	7 items: Who was/were told about the harassment	T1, T2, T3
Own reactions [58]	11 items: Behavioral and emotional reactions to the harassment, adapted	T1, T2, T3
Sexual harassment perpetration	6 + 5 items: verbal, visual, and physical sexual harassment perpetration	6 items at T1 and T2 and 11 items at T3
Sexual harassment witnessing	6 + 5 items: verbal, visual, and physical sexual harassment witnessing	6 items at T1 and T2 and 11 items at T3
Sexual harassment at school and in the class	2 items: Sexual harassment is seen as a problem at school and in the class	T1,T2,T3
Self-esteem [59]	1 item: self-esteem	T1, T2, T3
Children's self-efficacy scale [60]	4 items about self-assertiveness, eg. “Stand up for myself when I feel I am being treated unfairly”	T1, T2, T3
Children's hope scale [61]	6 items about agency and pathways, eg. “Even when others want to quit, I know that I can find ways to solve the problem”	T1, T2, T3
*Teacher reports*		
Demographic information	Gender, age, educational background, teaching experience, role at the school	T1, T2, T3
Knowledge/Awareness about sexual harassment in the class	6 + 5 items	T1, T2, T3
Class norms about sexual harassment	8 items: Different reactions that students might have to witnessing sexual harassment among peers	T1, T2, T3
Teacher’s perceptions of the seriousness of sexual harassment and bullying	6 items: How serious a threat to students’ well-being the teacher considers sexual harassment and bullying to be, respectively	T1, T2, T3
Teacher’s intentions to intervene [62]	7 items: Teachers’ intentions to intervene in sexual harassment between students, adapted	T1, T2, T3
Teacher’s efficacy for intervening [62]	14 items; teachers’ efficacy in intervening in sexual harassment between students, adapted	T1, T2, T3
School Personnel Barriers to Bystander Action [62]	5 items: Teachers’ perceived barriers to intervening in sexual harassment between students, adapted	T1, T2, T3
Perceptions of School Readiness [62]	12 items: Teachers’ perceptions of school readiness to work effectively against sexual harassment among students, adapted	T1, T2, T3
Teacher’s conceptualization of sexual harassment	Open-ended question	T1, T2, T3
Teacher’s ideas about why sexual harassment among students occurs	Open-ended question	T1, T2, T3
Teacher’s ideas for the prevention of sexual harassment among students	Open-ended question	T1, T2, T3
*School data*		
Average parental education level in specific schools	1 item on parents’ mean educational level on a 3-point scale: 1 = primary, 2 = secondary, 3 postsecondary	T1, T2, T3
School’s grade point average	1 item: The grade point average for the school	T1, T2, T3
Reports of sexual harassment	Filed reports of sexual harassment at the school	T1, T2, T3
Measures against sexual harassment at the class and school levels	Active measures taken against sexual harassment at the school	T1, T2, T3
Content of the school’s diversity and equal treatment policies	Analysis of any content of the school’s diversity and equal treatment policies that relates to sexual harassment	T1, T2, T3

^a^T1 = grade 4; T2 = grade 5, T3 = grade 6

Measuring sexual harassment among young people is sensitive. It is important that all instruments are developmentally- and age-appropriate. During the spring of 2019, we have developed a measure of sexual harassment to be used among children (ages 10–12 years). The measure consists of six items about physical, verbal, and visual sexual harassment at age 10–11 years; and an additional set of five items to measure physical, verbal, and visual sexual harassment at age 12 years. We ask about sexual harassment only among peers and at school. Other measures of sexual harassment used among older age groups [4, 5, 13, 18, 25, 26] have been used as inspiration when designing our measure. We have also consulted a group of experts (clinical psychologists, researchers, and school personnel) on child and adolescent development in general, and sexual harassment among young people in particular, in the development of our measure of sexual harassment. Both the student and the teacher questionnaires have been pilot-tested with children of the same age as the target group and 4–6 grade teachers, respectively.

### Procedure

Participant recruitment will be done in municipalities in the western part of Sweden, in and around the Gothenburg region. We will recruit classes in public and private (charter) schools in urban and non-urban areas by contacting school principals and school health service staff. Schools that teach grade 4 to grade 6 (Swedish 4th grade children are aged around 10) will be approached.

Data will be collected via questionnaires completed by participating children and teachers, and via public data registers and contacts with the school principal (e.g., school policies). Students will fill out pen-and-paper questionnaires during regular school hours. Classrooms will be set up in a way to ensure that students will be able to fill out their questionnaire privately. At or around the same time, the class’s main teacher will respond to the teacher questionnaire. We will send a questionnaire to the school principal via e-mail to obtain the school-level data. The data will be collected by the research team. The research team includes three senior developmental psychologists and one PhD student, who is a trained clinical child psychologist. Master’s students will also be part of the research team and participate in the data collection.

### Ethical considerations

The PRISE study has been approved by the Swedish Ethical Review Authority (reference number 2019–02755). Written informed consent will be obtained from the legal guardians of the participating children and from the participating teachers. Oral informed consent will be obtained from the children. Children will be orally informed about the research in a way that they can understand. School health staff will be informed about the study in advance so that they can take any action necessary. Children will be informed, orally and in the questionnaire, that they can get in touch with their local school health nurse or school counsellor if they feel the wish or need to do so. Participants may withdraw from the study at any time without risking any negative consequences. All data will be handled confidentially. All participating school classes will be offered an incentive corresponding to EUR 140 per year.

### Analysis

To answer Research question 1, we will mainly use descriptive statistics, including measures of central tendency and measures of variability. We will report on the percentage of students that report experiences of sexual harassment victimization, perpetration, and witnessing by gender for each time point. We will also report on teacher reports of sexual harassment among their students. Research questions 2, 3 and 4 will be answered using mainly multivariate regression analysis including structural equation modelling and growth analyses. Analyses will be both exploratory and confirmatory. Subgroup and multilevel analyses will be performed. We will control for the effects of other forms of peer harassment/victimization in the analyses in order to specify the unique effect of peer harassment that is sexual in nature. To answer the question of what the predictors of peer sexual harassment victimization, perpetration, and witnessing are during the transition from late childhood to adolescence (Research question 2), we will examine predictors at the school level (e.g., diversity and equal treatment policies), teacher/classroom level (e.g., teacher’s perceptions of the seriousness of sexual harassment and bullying), and individual level (e.g., pubertal development). Similarly, to answer Research question 3 (What are the developmental consequences of peer sexual harassment for different subgroups of children during the course of middle school?) we will examine outcomes at the school level (e.g., reports of sexual harassment), teacher/classroom level (e.g., norms/beliefs about students’ reactions to sexual harassment in the classroom), and individual level (e.g., self-esteem, externalizing symptoms, internalizing symptoms). We will examine the moderating effects of gender and age. Moreover, we will study the interrelationships among sexual harassment victimization, perpetration, and witnessing over time as part of answering Research questions 2 and 3. Concerning Research question 4 (What biological, psychological, social, and contextual risk and protective factors moderate the potential link between sexual harassment and its developmental consequences?), we will study the role of gender, pubertal development, own reactions to sexual harassment, child resilience, and teacher/school responses to sexual harassment as moderators of the links identified in the analyses conducted to answer Research question 3. Furthermore, the data collected from schools (e.g., reports of incidents related to sexual harassment) will be analyzed quantitatively and qualitatively (content analysis).

## Discussion

The described longitudinal research project, PRISE, which is based on a developmental-contextual framework [40], will enable the researchers to answer fundamental, unresolved questions about the early stages of sexual harassment among young people. The PRISE study will advance the literature by studying individuals during a key developmental transition (i.e., from childhood to adolescence) in their contexts and by using multiple reporters of and perspectives on sexual harassment at school. Specifically, the study will move beyond current knowledge by examining the nature of the problem of sexual harassment when it is assumed to typically begin. This, in turn, will help in identifying the young people most at risk of negative outcomes. At the same time, it will also provide knowledge about protective factors. In summary, the PRISE study will advance the very limited understanding of sexual harassment during an age period that is central in a person’s physical, sexual, and social development.

The PRISE study raises ethical questions that need to be addressed and handled carefully and sensitively. Ethical considerations are key given that the main participants are vulnerable (by being children) and that the instruments concern sensitive topics (e.g., peer victimization and perpetration). Although we have developed the questions about sexual harassment giving special consideration to the participants’ ages, there is still a risk that some of the children will feel provoked or uncomfortable in answering them. This may be particularly true for children who have been exposed to sexual harassment. Some researchers also describe what is referred to as the “question-behavior-effect” [63]; that behaviors that are asked about, especially risk behaviors, will increase as a result of asking about them. Taking these issues into consideration, we will collaborate with the school health staff at each school to ensure that they are available for students in need of support. We will also follow suggested guidelines when designing the questionnaire to counteract the question-behavior-effect. On the other hand, it should be taken into account that in order to counteract sexual harassment and its negative consequences during the transition from late childhood to early adolescence, we are in urgent need of empirical knowledge concerning this matter. We also believe that many children will appreciate participating in the project through being able to share their experiences and having their voices heard. Given the project’s societal benefits and that we will take action to avoid that any children getting hurt, we believe that the potential benefits of this project surpass its potential risks.

### Implications for policymaking and practice

With the advent of the #metoo-movement, it became clear that sexual harassment is a widespread, worldwide concern, that needs to be addressed on all levels of society. While children have the right to education, it also remains without doubt that sexual harassment interferes with this right [22]. Consequently, schools should have a strong impetus to create a safe environment for their students, free from sexual harassment. To be able to do so, more knowledge about the phenomenon, especially at the age when it typically begins, is urgently needed. Knowledge about when, where, why sexual harassment occurs and who are its victims can help delineate potential ways to act against it early in its development. The PRISE study will move beyond current knowledge by examining the problem of sexual harassment in the transition between late childhood and early adolescence, enabling a better understanding of its onset. It will use a longitudinal design, to identify both risk and protective factors for sexual harassment and its consequences. In addition, the project will examine sexual harassment from the perspectives of victims, offenders, and witnesses, as well as on multiple contextual levels (individual, classroom, and school), to enable a more ecological understanding of the phenomenon. Ultimately, the hope is that the project will help researchers, policymakers, and practitioners to develop, implement, and test interventions that can effectively combat a major, current societal challenge and adverse aspect of young people’s developmental ecologies.

## Data Availability

The dataset generated and analyzed during the current study is not publicly available due to its longitudinal nature and the sensitivity of the questions, but is available from the corresponding author on reasonable request.

## Abbreviations

DTE: Developmental Theory of Embodiment
EUR: Euro
PRISE: Peer Relations In School from an Ecological perspective
